# Assessment of medical waste generation, associated environmental impact, and management issues after the outbreak of COVID-19: A case study of the Hubei Province in China

**DOI:** 10.1371/journal.pone.0259207

**Published:** 2022-01-24

**Authors:** Jinquan Ye, Yifan Song, Yurong Liu, Yun Zhong

**Affiliations:** 1 School of Management, Nanchang University, Nanchang, 330031, PR China; 2 Ji luan Academy, Nanchang University, Nanchang, 330031, PR China; 3 School of Economics and Management, Nanchang University, Nanchang, 330031, PR China; Universita degli Studi del Molise, ITALY

## Abstract

COVID-19 greatly challenges the human health sector, and has resulted in a large amount of medical waste that poses various potential threats to the environment. In this study, we compiled relevant data released by official agencies and the media, and conducted data supplementation based on earlier studies to calculate the net value of medical waste produced in the Hubei Province due to COVID-19 with the help of a neural network model. Next, we reviewed the data related to the environmental impact of medical waste per unit and designed four scenarios to estimate the environmental impact of new medical waste generated during the pandemic. The results showed that a medical waste generation rate of 0.5 kg/bed/day due to COVID-19 resulted in a net increase of medical waste volume by about 3366.99 tons in the Hubei Province. In the four scenario assumptions, i.e., if the medical waste resulting from COVID-19 is completely incinerated, it will have a large impact on the air quality. If it is disposed by distillation sterilization, it will produce a large amount of wastewater and waste residue. Based on the results of the study, we propose three policy recommendations: strict control of medical wastewater discharge, reduction and transformation of the emitted acidic gases, and attention to the emission of metallic nickel in exhaust gas and chloride in soil. These policy recommendations provide a scientific basis for controlling medical waste pollution.

## Introduction

COVID-19 pandemic is threatening human health and has resulted in many indirect influences on the environment [1]. Among them are ecological restoration due to restrictions on human activities and the increase in domestic solid waste and electricity consumption due to non-contact lifestyles [2,3]. In addition to domestic waste, the rapid utilization of masks, protective clothing, and large amounts of other medical supplies has generated large amounts of infectious medical waste [4]. The disposal of these medical wastes can cause several environmental hazards, which mainly include pollution of the atmosphere, waters, and soil [5]. Due to the lack of foresight and preparation for epidemics, excess low-risk medical waste is often disposed of at domestic waste standards [6], which further aggravates the impact of medical waste on human health and the ecological environment.

Due to the rapid spread of the pandemic, the resulting medical waste known for its long-term strong infectivity needs to be disposed of as soon as possible [7,8]. Medical waste is of great concern due to its potential harm to human health and the environment [9]. The incineration of medical waste produces a variety of harmful gases, and these gas mixtures can cause varying degrees of pollution to the air, water, and soil [10]. With the rapid increase in the number of confirmed cases, the risks of medical waste disposal and the subsequent environmental impacts are rapidly increased [11]. Therefore, it is important to estimate the amount of additional medical waste that would be generated by the pandemic and the amounts of contaminants it could produce. This can provide perspective and data to support environmental recovery in the post-pandemic era [12].

Research related to medical waste focuses on the evaluation of medical waste disposal technologies, economic benefits of medical waste disposal, medical waste production and composition management methods [13]. Earlier works on the environmental impact of COVID-19 focus on environmental recovery from reduced human activities, increased solid waste from non-contact lifestyles and disposal of plastic waste from the pandemic [14,15]. The above-mentioned works illustrate that many scholars are concerned about the huge environmental impact caused by waste generated during the pandemic [16–18], although to our knowledge, only some studies have reported on the quantification and environmental impact of COVID-19 medical waste [19,20]. The prerequisite for assessing the environmental impact of incoming medical waste from an pandemic is to reasonably estimate the medical waste production. The present means of predicting/ estimating medical waste production are mainly gray prediction models, field survey methods, simple linear regression methods, and empirical estimation methods, and each of these survey methods have many advantages and shortcomings. Therefore, exploring the means to estimate the amount of medical waste generated by COVID-19 and assessing its environmental impact is an urgent issue to be addressed.

In this study, first, the annual production of medical waste in Hubei province, China, was obtained by empirical calculation method and formula. Second, the actual amount of medical waste generated in a month was calculated based on the ratio of total hospital visits in that month to total hospital visits in that year. Then, the experimental results of various existing time series forecasting models were compared, and the long short-term memory (LSTM) model was selected to construct a counterfactual forecasting framework for medical waste under no pandemic conditions. By comparing the prediction results with the actual medical waste generation, the amount of additional medical waste after the occurrence of COVID-19 pandemic in the Hubei Province was calculated. Finally, the environmental impact assessment was carried out by estimating the difference of the composition and disposal of the increased medical waste under different scenarios. Four scenarios were assumed in this study, which are Business as Usual (BAU), Complete Pyrolysis (CP), More Pyrolysis (MP), and More Steam Sterilization (MS).

The rest of the paper is organized as follows. Section 2 describes the study objectives, methodology, and data sources. Section 3 reports the findings of the study and the analysis of the results. Section 4 presents the conclusions and further policy implications of this study. Section 5 discusses the limitations of the study.

## Methods and data

### Research subjects and scope

**Fig 1:**
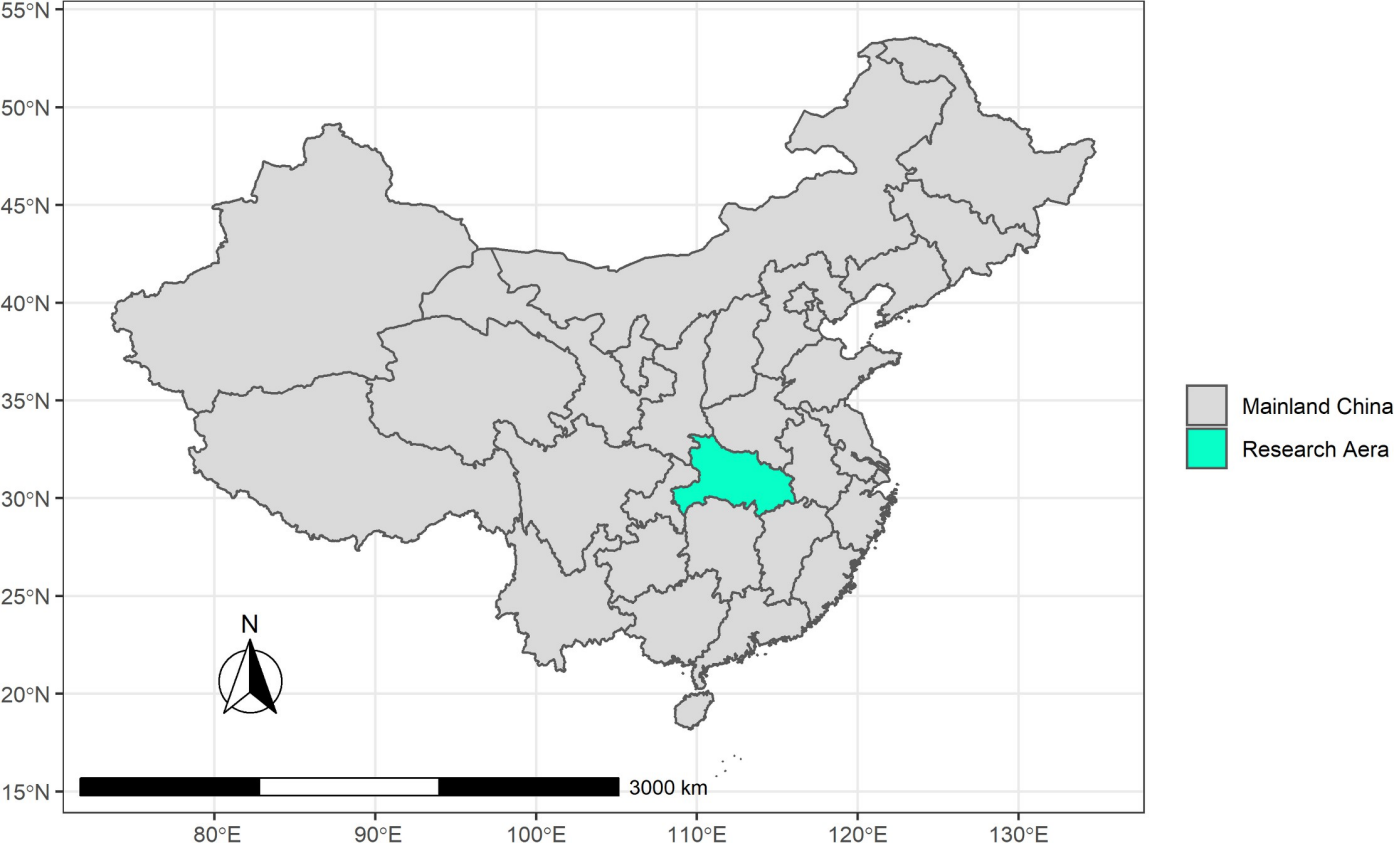
Map of Hubei Province.

The Hubei province is the epicenter of COVID-19 in China, and consequently the region producing the largest amount of medical waste [21]. Therefore, it was chosen as the subject of the study (Fig 1). According to the pandemic data published by Health Commission of Hubei Province, the pandemic in the Hubei Province mainly occurred at the end of January 2020 and lasted till the end of April 2020. Therefore, this paper focuses on the pandemic medical waste production in the Hubei Province from late January to the end of April 2020 and its impact on the environment.

### Calculation of annual production of medical waste

At present, the calculation methods of medical waste production mainly include field survey method and empirical estimation method [22,23]. The field survey method includes selecting several representative medical institutions in a certain area by random sampling, and then investigating the medical waste production of these medical institutions to obtain the basic situation of medical waste production [24]. However, this method is time-consuming, more expensive, and is not universally applicable. The empirical estimation method generally uses internationally accepted empirical formulas. In this study, the quantity of medical waste is calculated from the values of the variables of number of visits, bed utilization, and number of beds [25,26]. Therefore, the study implements the empirical estimation method to calculate the annual production of medical waste in the Hubei Province from 2014 to 2019, and these historical data are used as a basis to predict the medical waste production for each month in 2020.

There are various factors that affect the annual production of medical waste. Many researchers have conducted in-depth studies using regression models, in which the main influencing factors are the level of education, living standards of the population, level of economic development, number of beds in medical institutions, utilization rate of beds, level of medical services, number of visits [27–30]. It was found that the number of beds in medical institutions, the bed utilization rate, and the number of visits were the most important factors affecting the annual production of medical waste [31]. Therefore, in this study, the annual medical waste production in the Hubei Province was calculated based on the above factors for each year by applying the empirical formula Q, and the calculation formula as follows.


Q=365BPM+NS (1)

Medical waste in the Hubei Province for the calendar year consists of two parts. the outpatient department medical waste, and the inpatient department medical waste. In Eq 1, B denotes the number of beds in all medical institutions in the Hubei Province in a given year, P is the bed utilization rate of that year, and M indicates the average daily amount of medical waste generated per unit bed. N is the number of visits to all medical institutions in the Hubei Province in a given year, and S is the average amount of medical waste generated per unit visit per day.

### Estimation of monthly production of medical waste

According to the objective of this study, it is necessary to calculate the monthly medical waste production in Hubei Province in previous years and then use it as a basis to predict the monthly medical waste production under normal conditions in 2020. Although the number of beds, bed occupancy rate, and number of visits to medical institutions in the Hubei Province per month are not officially published, studies have shown that there is a highly positive linear relationship between the monthly medical waste production and the total number of visits to hospitals [32]. Therefore, in this study, the ratio of the total number of hospital visits per month to the total number of hospital visits in the Hubei Province in that year is used as the weight, and then the calculated values of the above annual medical waste production are multiplied by the weights of the corresponding months to obtain the monthly medical waste production as *q*_*i*_. The specific calculation formula is as follows.


$$q_{i}=Q\cdot \omega_{i}$$ (2)

$$\omega_{i}=\frac{n_{i}}{\sum_{i=1}^{12}\;n_{i}}$$ (3)

where, *ω*_*i*_ is the ratio of the total number of hospital visits per month to the total number of hospital visits in that year, and *n*_*i*_ is the total number of hospital visits in month *i* of a year in Hubei Province.

### Counterfactual predictions for medical waste

Based on the time series data estimated in the previous section, this section constructs counterfactual forecasts for the year 2020 without the occurrence of the pandemic. There are multiple prediction models to choose from for the prediction of time series data. Considering the limitation of sample size and the accuracy of prediction, this paper uses several models for prediction simulation and validates the set models by using various indicators. Finally, the LSTM model is selected to predict the amount of medical waste generated from January to April 2020. A comparison of the various predictive models is provided in Table A in S1 File.

#### Long short-term memory neural network

LSTM network is a special type of Recurrent neural network (RNN) that solves the problem of long-range dependencies in data by capturing multiple aspects of past information through multiple network layers. In econometrics, LSTM provides a new tool for dealing with time series data [33]. Currently, LSTM has been applied to prediction scenarios stock selection and forecasting [34] and solar activity prediction [35]. As a variant of RNN, LSTM has a neural network repetition chain structure. With a repetition unit of not just one but four internal network layers, LSTM network is able to capture long short-term memory.

LSTM solves the very streamlined form of the long dependency problem in RNN networks. In this network, a brief LSTM memory transfer is given by *c*^*t*^, the *h*^*t*^ is completed, and its relation to the output result *y*^*t*^ is expressed by the following equation.


$$c^{t}=z^{f}⊙c^{t-1}+z^{i}⊙z$$ (4)

where *c*^*t*^ represents the long-time part of the selective memory, the *z*^*f*^ serves as forget gate to control the previous state of *c*^*t*-1^, *z*^*i*^ represents the memory gate that is retained, and *z* is the current information scaled by the tanh-function.


$$h^{t}=z^{o}⊙\text{tanh}\left(c^{t}\right)$$ (5)

*h*^*t*^ represents the short-time memory part from the current output of the gate *z*^*o*^ and the long-time memory of Hadamard Product after tanh activation.


$$y^{t}=\sigma \left(W^{\prime }h^{t}\right)$$ (6)

*y*^*t*^ is the final output result, and similar to RNN, the output result is often ultimately obtained by the difference between the weight matrix and the obtained variation *h*^*t*^ after Sigmoid activation. To ensure the reliability of the prediction model selection in this study, Prophet, a seasonal-Auto Regressive Integrated Moving Average (ARIMA) model is used to compare with the LSTM time series prediction model, and its results are reported in the S1 File.

### Scenario assumptions for environmental impact assessment

We used a scenario-based approach to make assumptions about the composition and disposal of the estimated increase in medical waste due to COVID-19 outbreak. This will be used to conduct an environmental impact assessment. Pandemic medical waste differs from normal medical waste in two ways: 1. The nature of the waste differs: due to the infectious nature of COVID-19, and 2. The waste disposal method is different. The net value of medical waste estimated by the "Estimation of monthly production of medical waste" section was considered as infectious waste in this study [36]. Due to the lack of relevant data, our study uses the assessment data of typical medical waste as a substitute. Therefore, we used Jingmin et al. [37] proposed environmental assessment data for potentially infectious waste (Details are shown in the S1 File). According to government information [38], due to the surge of medical waste, almost all of the waste will be disposed of using the incineration method. Accordingly, the assumptions of following scenarios were made [39].

- Business as usual (BAU)
  In the BAU scenario, we consider the disposal of medical waste as a continuation of the previous approach. According to relevant reports [40], as of the end of December 2019, the centralized medical waste disposal in the Hubei Province has been licensed with a total capacity of 63,000 tons/year, 61% of which adopts high-temperature incineration treatment process and the remaining 39% adopts autoclave steam sterilization treatment. In view of this scenario, our study assumes that the pandemic in the Hubei Province adds medical waste (M = 0.5), and 61% of the medical waste is disposed of by high-temperature incineration and 39% by autoclaving.
- Complete pyrolysis (CP)
  In the CP scenario, we refer to the study by 28. To expand the waste disposal volume, it is assumed that the Hubei Province will adopt complete pyrolysis for waste disposal. According to this, all medical waste will be disposed of by high-temperature pyrolysis.
- More pyrolysis (MP)
  In the scenario where pyrolysis is preferred, we assume that pandemic waste disposal is prioritized by disposal volume [41]. Due to the large amount of medical waste due to the pandemic, the pressure of waste disposal is increased, which results in increase of the proportion of pyrolysis waste. Here, 80% of the waste will be pyrolyzed at high temperatures and the remaining 20% will be sterilized using autoclaving.
- More steam sterilization (MS)
  In the scenario where steam disinfection is preferred, we assume that outbreak waste disposal is prioritized in terms of infection risk reduction and environmental protection. Steam disinfection method disinfects medical waste in the presence of infectious agents by degrading proteins and destroying microbial tissues. During this process, no harmful gases are released [42]. In the MS scenario, we increase the percentage of steam disinfection method in BAU such that 60% of medical waste is disinfected by steam disinfection and 40% by pyrolysis methods.

### Related data sources

#### Annual production related data sources

In this study, we obtained the statistics of the number of beds, bed utilization rate, and attendance of medical institutions in the Hubei Province from 2008 to 2019 by reviewing relevant information from the National Bureau of Statistics (China) (as shown in Table 1).

**Table 1:** Indicators related to medical institutions in the Hubei Province over the years.

Year	Bed numbers/10000	Bed utilization rate %	Visits (Billion times)
**2008**	16.73	87.3	1.44
**2009**	18.72	92.7	2.18
**2010**	20.04	96.1	2.39
**2011**	22.40	98.7	2.68
**2012**	25.30	99.3	3.06
**2013**	28.82	96.5	3.21
**2014**	31.75	96.1	3.45
**2015**	34.31	92.4	3.48
**2016**	36.06	92.0	3.55
**2017**	37.62	92.7	3.56
**2018**	39.35	92.7	3.51
**2019**	40.33	92.3	3.54

Data source: National Bureau of Statistics (NBS) (China).

In calculating the annual production of medical waste, the daily production of medical waste per unit bed M in Eq (1) and medical waste production per unit visit S are not directly available through official websites. For the S value, we searched the relevant literature in China and abroad [43]; the daily medical waste generation per unit visit was found to be 0.03–0.05 kg, and the value positively correlated with the economic development level. Therefore, based on the level of economic development in China, this study considers the average value of 0.04 kg/visit/day. For the M value, there are large differences among different countries and minor differences among different regions of the same country [44]. According to a study by domestic scholars [23,45,46], the medical waste generation rate in Gansu Province in 2010 was 0.59–0.79 kg/bed/day, and the medical waste generation rate in 2014 in the Enshi Prefecture, Hubei Province was about 0.37 kg/bed/day. The average medical waste generation rate in the Hubei Province in July 2016 was about 0.5 kg/bed/day. Given the development of economic conditions, infrastructure and medical services in the province in recent years, this study sets the medical waste generation rate at 0.5 kg/bed/day. A sensitivity analysis was also performed on the M value, which is the daily generation of medical waste from hospital beds, and the results are presented in the S1 File.

#### Monthly production related data sources

**Fig 2:**
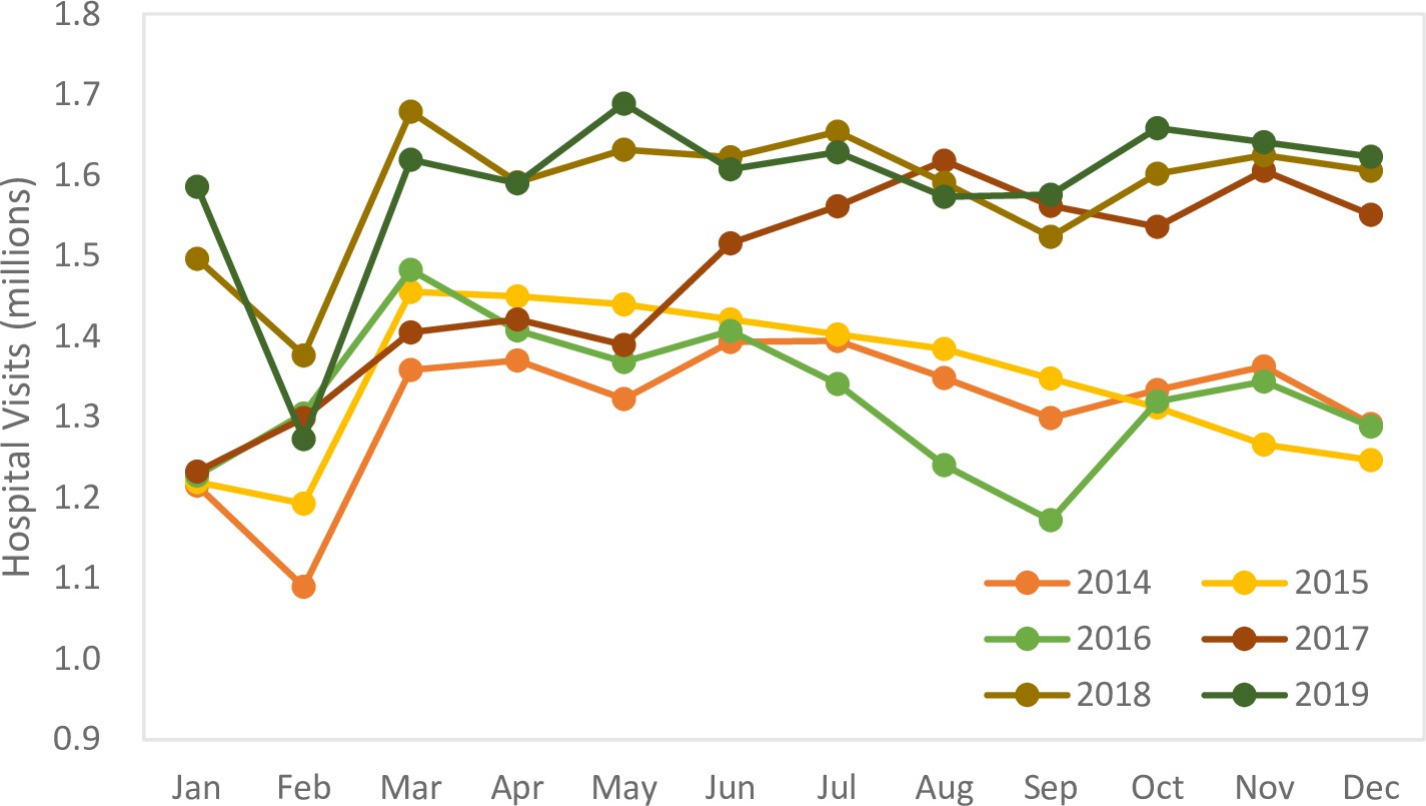
Graph depicting the hospital visits by month in the Hubei Province from 2014–2019 (million visits).

By reviewing the relevant data from the National Health and Wellness Commission of the People’s Republic of China, we obtained all hospital visits per month in the Hubei Province from 2014–2019 as shown in Fig 2.

The percentage of each month was calculated based on the total number of hospital visits in each month from 2014–2019 in the Hubei Province, which is the weight of medical waste production in each month to the total medical waste production in that year.

#### Environmental impact-related data sources

According to domestic and international studies on medical waste disposal, different disposal methods may be suitable for different categories of medical waste, and the disposal technology for medical waste is mainly divided into two types, incineration and non-incineration. The most common method of the latter type is autoclaving [47].

Medical waste disposal produces a mixture of hazardous gases, including carbon monoxide, sulfur dioxide, nitrogen oxides, fluoride, various metals and their compounds, dioxins, and other volatile organic compounds [48]. Among them, mercury in exhaust gases not only pollutes the atmosphere, but also enters the water and soil with the air flow, and thus degrades water sources and inhibits plant growth. The toxicity of dioxins is much higher than that of other toxic gases, and dioxin concentrations in flue gases from medical waste incineration are significantly higher than those from domestic waste incineration [49]. Sulfur dioxide in exhaust gases also contributes to atmospheric acidification, which in turn can lead to high-risk natural hazards such as acid rain [50]. Medical waste that is randomly disposed into rivers and lakes can easily lead to a decrease in lake size, changes in the acidity and alkalinity of water bodies, and the death of a large number of aquatic organisms [51]. The infiltration of many harmful substances in the soil may change its pH, reduce its fertility, and affect the survival of soil microorganisms and plant growth [8,52,53]. The sources of toxic compounds, their hazards and their emission limits are explained in detail in Table D in S1 File.

Earlier research by domestic and foreign scholars reported that a variety of hazardous substances are produced after medical waste disposal, and the amount of production depends on the employed disposal technology [54]. In this study, the main hazardous substances produced by two common disposal technologies were obtained by reviewing the relevant literature [37].

## Results and discussion

### Estimated monthly production of medical waste

**Fig 3:**
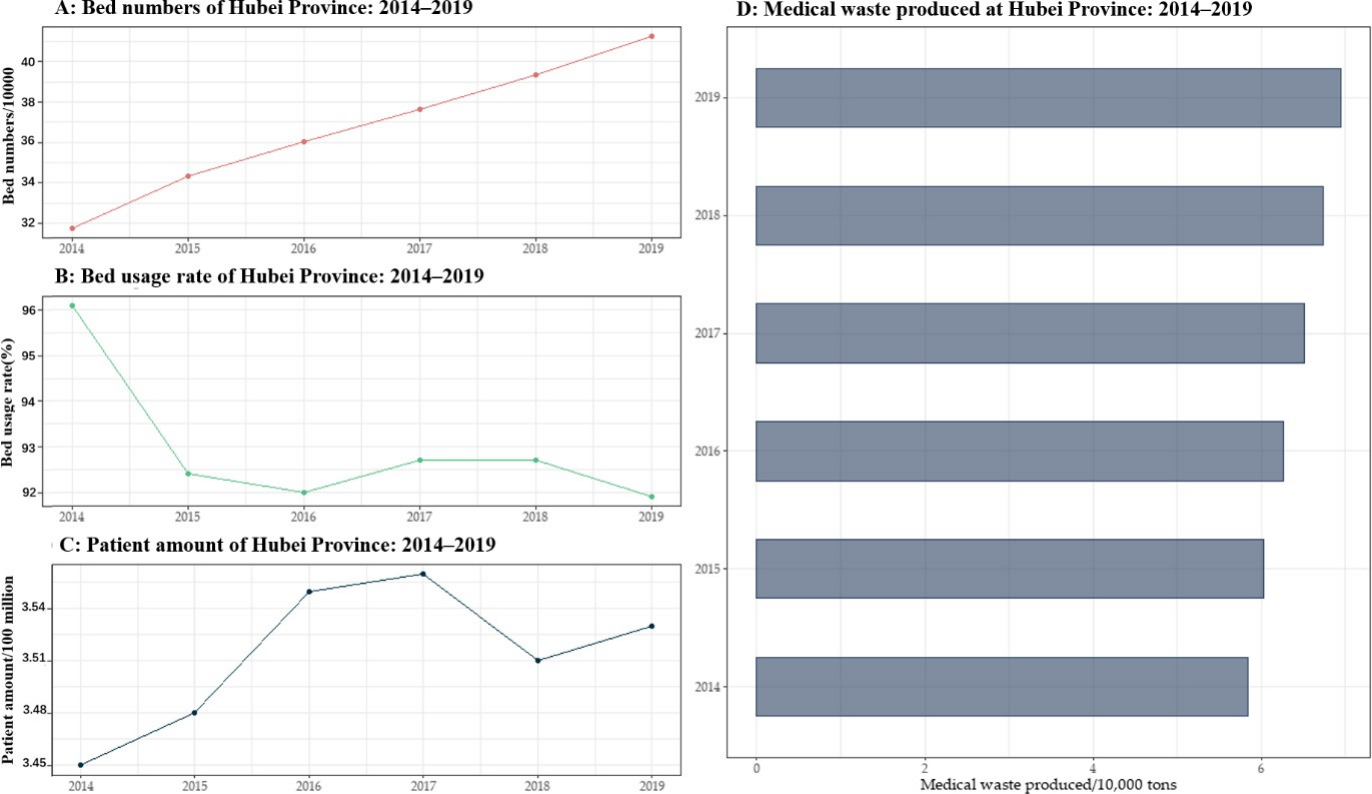
Graphs exhibiting the annual production of medical waste in the Hubei Province, 2008–2019.

The monthly production of medical waste in the Hubei Province was calculated based on the annual production of medical waste and the weights of each month. We first calculated the annual production of medical waste from 2014 to 2019 by using Eq (1) and the relevant was data collected (as shown in Fig 3).

**Fig 4:**
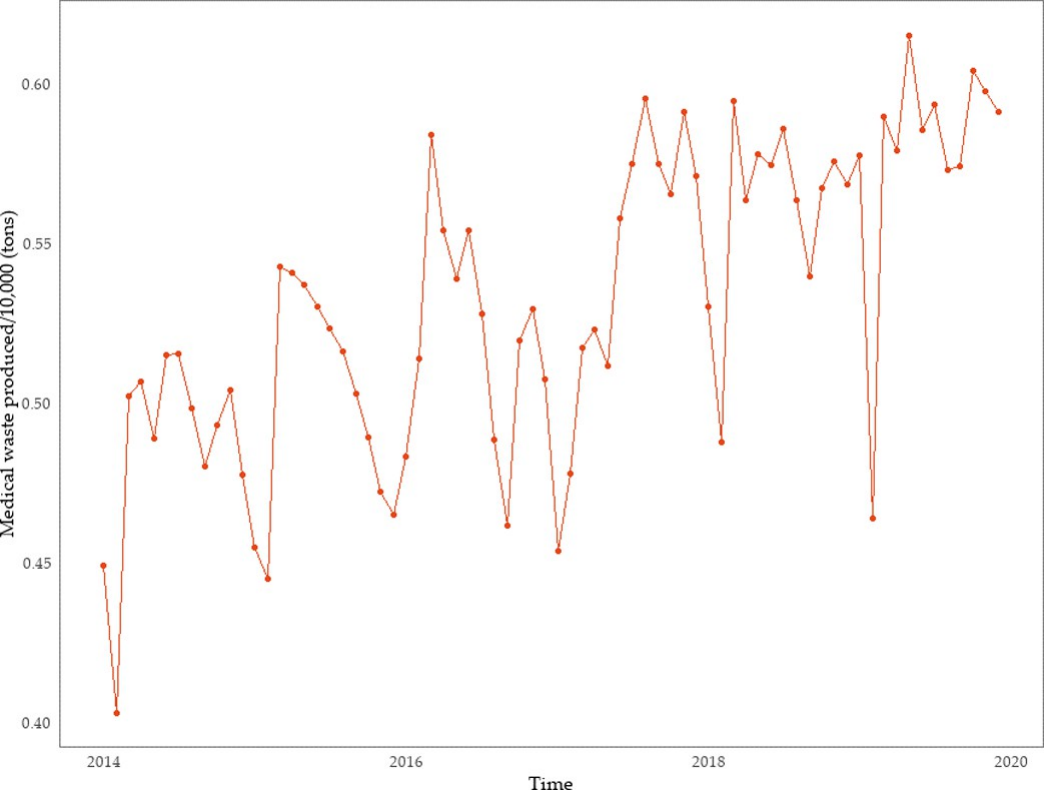
Plot showing the monthly production of medical waste in the Hubei Province, 2014–2019.

Fig 3 depicts the trend of medical waste, patient visits, bed utilization rate, and number of beds in the Hubei province from 2008–2019. Plot A represents the change in the number of hospital beds, where the bars represent the number of beds in tens of thousands, which is seen to increase over time. Plot B represents the annual bed utilization rate, which is shown to fluctuate in the graph. Plot C depicts the trend of growth in the number of patient visits. Plot D represents the estimated annual medical waste generated. It is initially recognized from Fig 3 that the number of consultations and hospital beds in Hubei province show an increasing trend year after year, which is in line with previous studies [55], and such an increase may be caused by the increasing resident population and the growing industrialization. [23,56–58]. In this study, the above annual production data and the weights of each month of the corresponding year were used to calculate the monthly medical waste generation in the Hubei Province from 2014–2019 (as shown in Fig 4).

From Fig 4, it is seen that the lowest peak of monthly medical waste generation in the Hubei Province mainly occurs in February each year, and the highest peak in March each year (sometimes from May–August). The main reasons for this pattern are as follows: (1) The spring festival usually falls in February. Generally speaking, most Chinese people tend to avoid visiting medical institutions and other similar places during the most important traditional festival; (2) March follows right after the spring festival, a time when more people are willing to go out, which includes visiting hospitals for diagnosis and treatment; (3) The Hubei Province has a relatively developed tourism industry, and the May–August period is the first month after the Chinese New Year. (4) The tourism industry in the Hubei Province is relatively well developed, and May–August is the peak period for tourism, which increases the flow of people, and possibly the number of patients.

### Medical waste monthly production forecast

The LSTM Model was used to obtain the counterfactual prediction of the scenario where there was no COVID-19 pandemic in 2020. The medical waste generation rates of 6765, 5838, 6864, and 6777 tons from January to April 2020 were obtained for the case of medical waste generation rate of 0.5 kg/bed/day.

### New medical waste production from the outbreak

According to the pandemic data released by the Hubei Provincial Health and Wellness Commission, the pandemic broke out on January 23, 2020, and ended on April 28, 2020. According to the Hubei Provincial Department of Ecology and Environment, from January 23 to April 28, the Hubei Province safely disposed of a total of 24,357.99 tons of medical waste, which can therefore be inferred as the medical waste generated by the hospitals during the pandemic period in the Hubei Province.

To calculate the additional medical waste production during the pandemic period compared to that of the normal period, this study needs to first calculate the medical waste production under normal conditions in the Hubei Province from January 23 to April 28, 2020. According to the medical service data published by the National Health and Wellness Commission of the People’s Republic of China, under normal conditions, the number of hospital beds, bed occupancy rate, and the number of attendances on each date of the same month vary so little that it could be neglected. Therefore, under the empirical estimation method, this study assumes that the daily medical waste production in Hubei Province is the same in each month under normal conditions. Based on the predicted medical waste production in the Hubei Province from January to April 2020 (M = 0.5), the medical waste production from January 23 to April 28, 2020 under normal conditions can be calculated (as shown in the Table 2).

**Table 2:** Predicted production of medical waste under conventional conditions in the Hubei Province.

Time	Medical waste(ton)
1/23-1/31	1964
2/1-2/29	5838
3/1-3/31	6864
4/1-4/28	6325
Sum	20991

Based on the actual production value of medical waste from the pandemic in the Hubei Province and the total normal production from Table 2, we can obtain the net production value of new medical waste of 3366.99 tons, which is 16.04% higher than that under the normal conditions in the same period. For reference, this study also predicts the medical waste generation on normal conditions based on 0.4 kg/bed/day or 0.6 kg/bed/day. The result is presented in the S1 File.

### Scenario analysis

**Fig 5:**
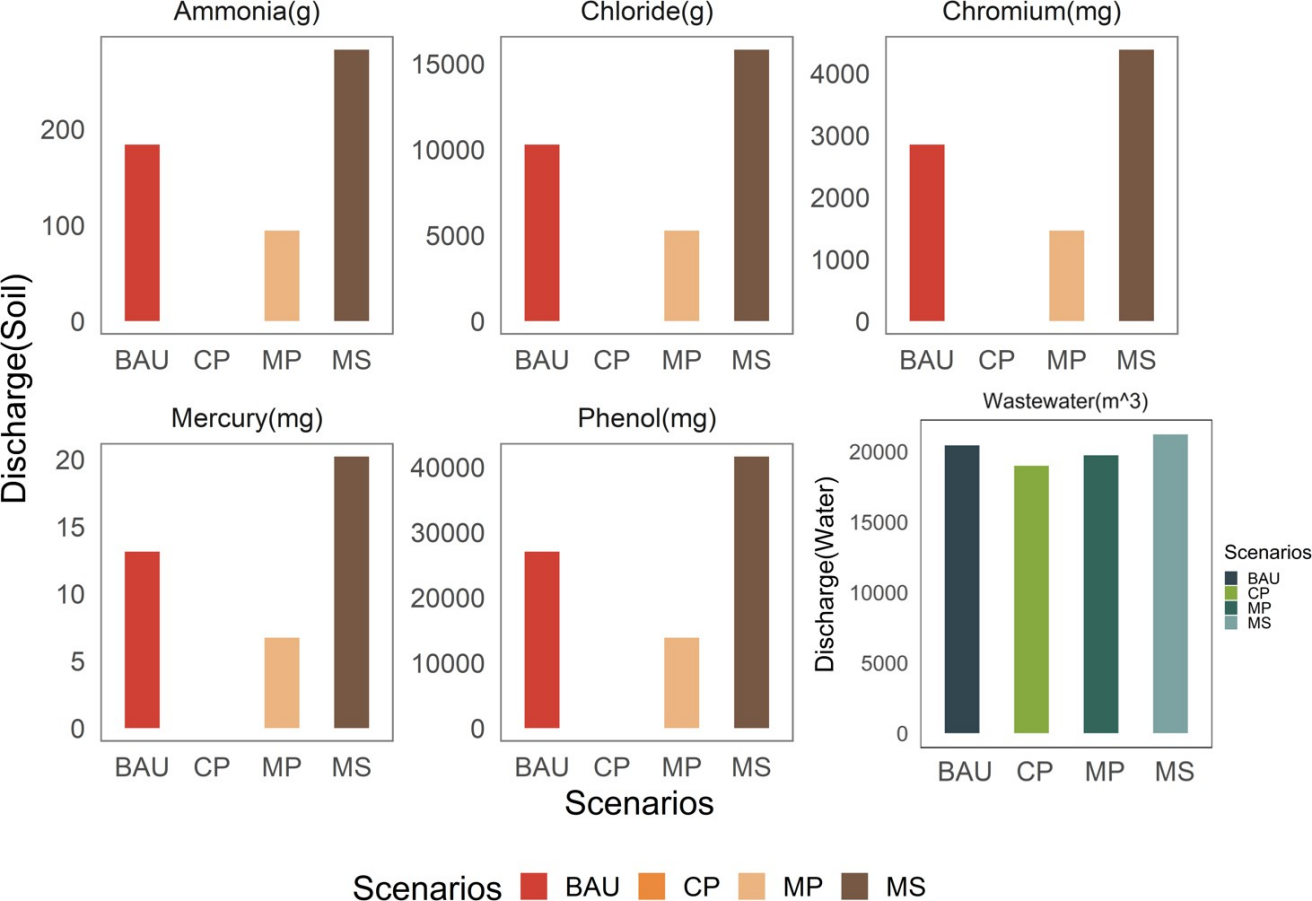
Bar plots showing the wastewater and waste production under four scenarios.

**Fig 6:**
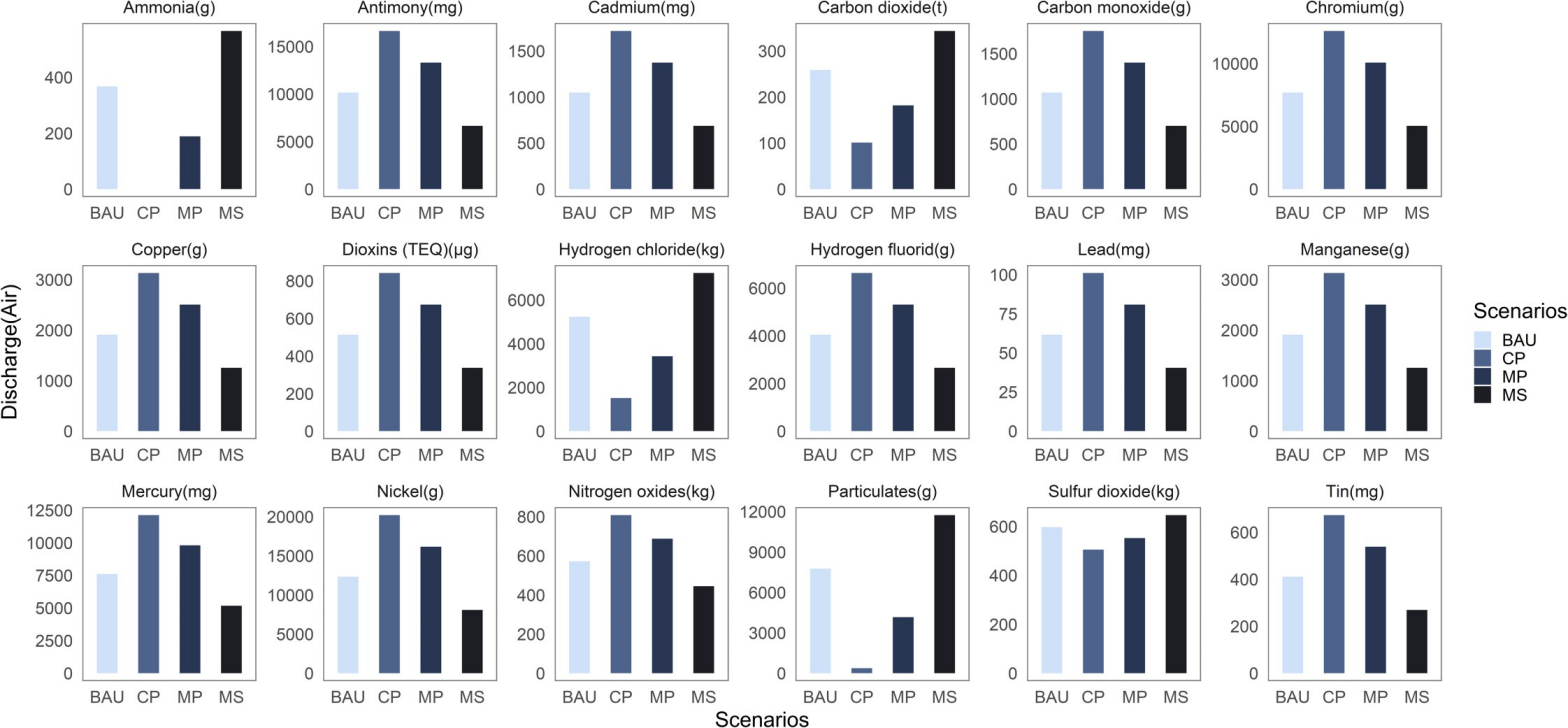
Bar plots depicting the exhaust gas production under the four scenarios.

For the environmental impact of pandemic medical waste, this study set four scenarios and calculated the impact situation of medical waste on environmental factors under various scenarios by adjusting the application ratio of two disposal technologies, and the results are shown in Figs 5 and 6.

From Fig 5, it is seen that the order of the magnitude of wastewater and sludge emissions under the four scenarios is MS>BAU>MP>CP, which implies that the high-temperature incineration method can reduce the impact of medical waste disposal on the water and soil environments. Especially for the discharge of wastewater, which contains many harmful substances, such as chloride, fluoride, sulfur dioxide, mercury, and other heavy metals. The amount of discharged wastewater is quite huge in all four scenario assumptions, and hence, the government should manage wastewater generated during medical waste disposal. It should strictly control the discharge of such wastewater and the emission standards of the concentration of various chemical substances contained in it, and simultaneously enhance the supervision and subsequent punishment of the medical waste disposal industry to ensure that the harm caused by wastewater to human health and ecological environment is minimized.

Further, it is seen from Fig 5 that chloride emissions are the highest among the waste materials discharged into the soil, which exceeds the sum of emissions of other harmful substances. Excessive chloride in the soil is likely to cause soil acidification, salinization, and even soil erosion. Therefore, for countries and regions with serious pandemic, local governments should strengthen the control of chloride content from medical waste disposal, and devise appropriate methods to collect and reuse the chloride to avoid environmental pollution caused by large amount of chloride discharge into the soil.

Fig 6 depicts that the order of magnitude of most of the exhaust gas emissions in the four scenario assumptions is CP>MP>BAU>MS. Therefore, steam sterilization method produces less exhaust gas than high-temperature incineration method, although it produces more sulfur dioxide, hydrogen chloride, and carbon dioxide gases. Among the harmful exhaust gases emitted, hydrogen chloride gas has the highest emissions. Sulfur dioxide and hydrogen chloride are easily combined with water vapor when emitted directly into the atmosphere, which can potentially form acid rain. This can have an extremely negative impact on human health and the ecological environment. Furthermore, carbon dioxide accumulates in the atmosphere, which tends to create a greenhouse effect. Therefore, countries with serious pandemic should monitor the concentration of acid gases generated by medical waste disposal in real time, organize experts and scholars to discuss and study this issue, and use cost-effective means to convert these acid gases into harmless gases.

Additionally, the emissions contain many heavy metals, among which content of nickel was the highest and lead content was the lowest (Fig 6). Nickel and its compounds emitted into the atmosphere can easily form dust and affect the growth of plants when they land in the soil, and through certain chemical reactions they can also produce various carcinogenic substances. Therefore, among the many metallic substances contained in exhaust gas, the government should pay special attention to the emission of nickel metal, improve relevant laws and regulations at the earliest, and improve medical waste disposal technology. Especially for countries with serious pandemic, such as the United States [59], Brazil [60], and India [61], the government should take effective measures to reduce the large amount of nickel particles generated by medical waste disposal.

**Fig 7:**
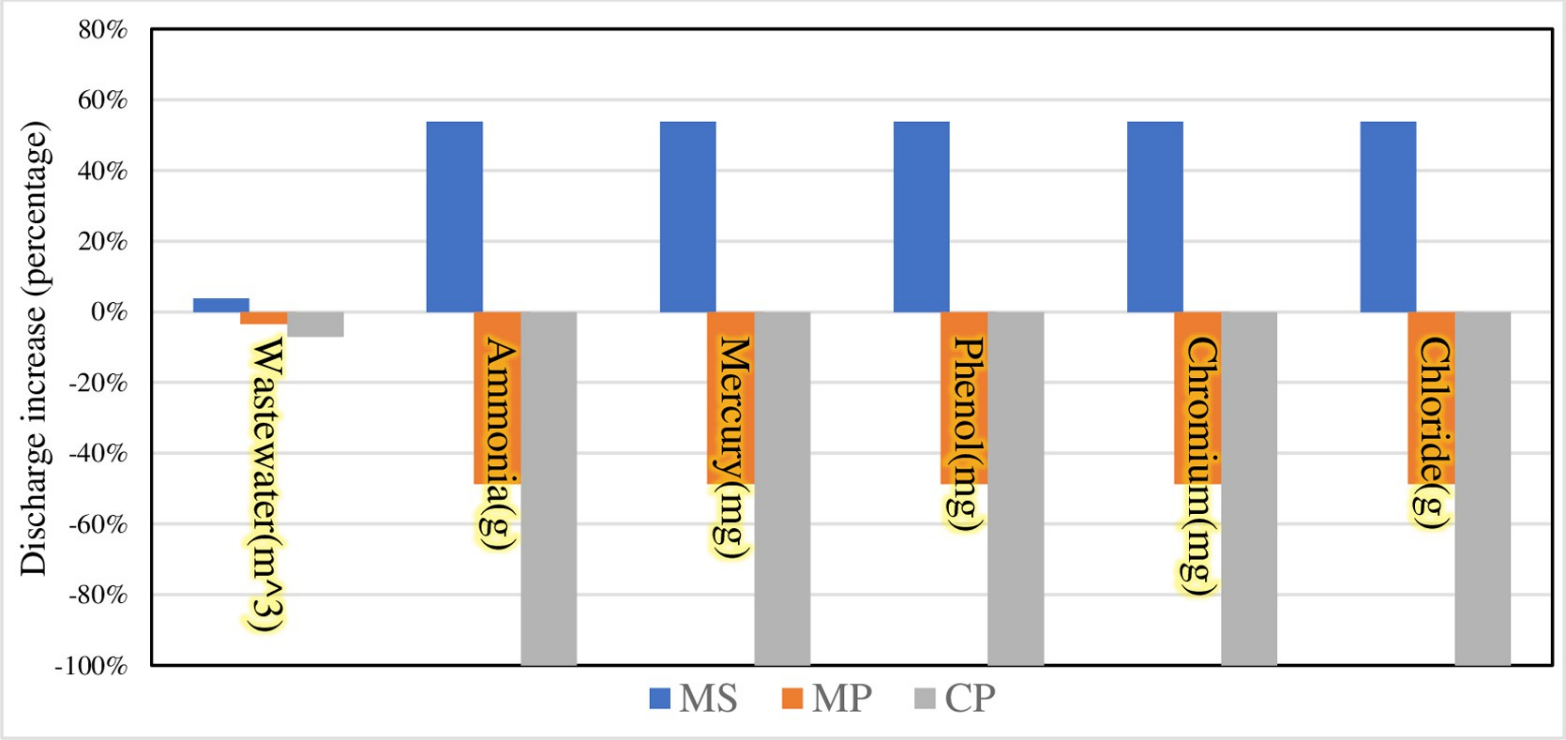
Bar graph depicting the percentage increase in wastewater and waste production compared to the BAU scenario.

**Fig 8:**
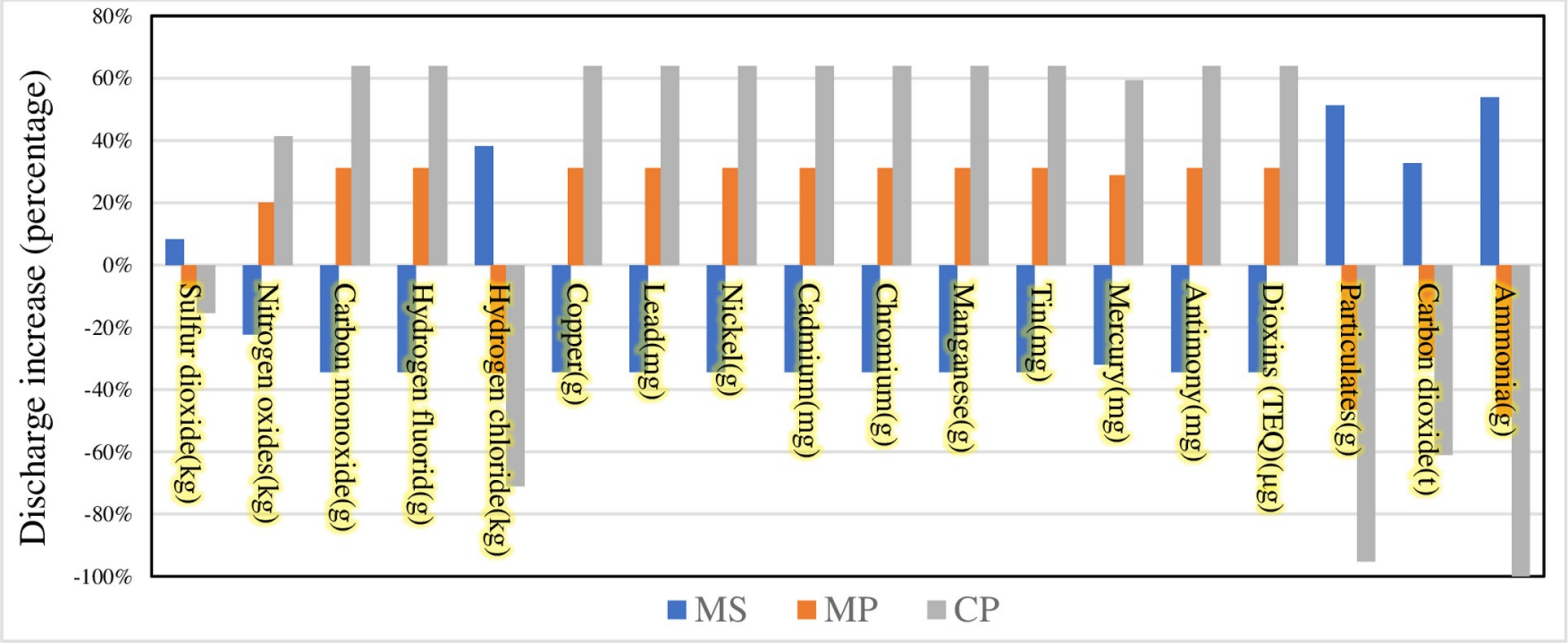
Bar plot showing the percentage increase in exhaust gas production compared to the BAU scenario.

Finally, we compare the remaining three scenarios with the BAU scenario to explore the proportional change in the impact of different scenarios on environmental factors compared to that of the BAU scenario. The results are shown in Figs 7 and 8.

As shown in Fig 7, the MS scenario is compared to the BAU scenario, where both wastewater and waste are increased in the MS scenario. During the COVID-19 pandemic, the government needs to use more steam sterilization to treat medical waste to reduce the risk of infection due to the need to prevent and control the pandemic. Compared to the BAU scenario, the MP and CP scenarios result in different degrees of reduction in wastewater and waste generation. The most significant reduction in emissions is the CP scenario. In terms of direct soil emissions resulting from medical waste disposal, pyrolysis is environment friendly and sustainable due to its clean and safe characteristics [62], which allows for efficient treatment of medical waste. However, pyrolysis is preceded by pretreatment of medical waste, a process that entails significant energy costs [63]. Therefore, during the pandemic, the government needs to increase its support to relevant companies to help them improve their equipment and their processes, and if necessary, to subsidize energy.

The changes in direct air emissions from the different scenarios of medical waste disposal are then compared (Fig 8). The MS scenario reduces most of the emissions compared to the BAU scenario. For example, the emission reductions for Nitrogen oxides, Carbon monoxide, Hydrogen fluorid, Hydrogen chloride are in the range of 20 to 40%. But for ammonia, ammonia gas, mercury, phenol, chromium, and chloride, their emissions are significantly increased. As organic compounds such as phenol are hazardous to humans, they may pose a health risk to the people involved in handling medical waste [64]. Therefore, governments need to regulate medical waste disposal methods during pandemic and pre-treatment of different medical wastes can effectively reduce harmful emissions. MP and CP scenarios increase the emission of heavy metals such as copper, tin, mercury and dioxins compared to BAU scenario. The first is that these heavy metals are emitted into the atmosphere in gaseous or in solid form adsorbed on fly ash, which has environmental biotoxicity and bioaccumulation, and poses a serious threat to ecology and human health. Second, the dioxins emitted into the atmosphere are transferred to the soil and easily adsorbed to the organic matter of the surface soil. Dioxins from medical waste disposal can have negative effects on vegetation and human body. Therefore, under the MP and CP scenarios, the government first needs to focus on monitoring the levels of dioxins and heavy metals in the soil around the emission sources. Third, relevant government authorities need to strengthen the supervision of medical waste disposal enterprises and update the medical waste disposal facilities and management methods of old enterprises, to minimize the harm caused by medical waste disposal to the environment.

## Limitations

This study tried to restore the environmental impact caused by medical waste disposal during COVID-19 to the best possible extent. However, due to the difficulty in obtaining primary data, the study uses the assessment data of typical medical waste as a substitute. In the assumption scenarios, we tried to quantify the impact of the disposal of COVID-19 medical waste as close as possible to real-life scenarios, although due to the complexity of the realistic recycling process, it was difficult to cover all hypothetical situations.

## Conclusions

In this study, we found that at a medical waste generation rate of 0.5 kg/bed/day, COVID-19 resulted in a net increase in medical waste volume of about 3366.99 tons in the Hubei Province. The possible environmental impacts under different disposal methods were modeled to provide a reference for medical waste disposal during a pandemic. the MS scenario was able to reduce most of the waste gas emissions compared to the BAU scenario, with a reduction of between 20% and 40%. The disadvantage is that the MS scenario increases the amount of wastewater and waste generated. On the contrary, the MP and CP scenarios compared to the BAU scenario lead to different reductions in wastewater and waste generation. The disadvantage of these two scenarios for medical waste disposal is that they increase the emission of heavy metals and dioxins. This provides a policy basis for how countries or regions with severe pandemic situations can safely and effectively handle medical waste.

## Supporting information

S1 File Supporting material on scenario analysis, toxic inventory, and comparison of time series prediction models. All publicly available data are in tabular form in this document.
(DOCX)

S2 File (PDF)
