# Supplementary material for: Assessment of medical waste generation, associated environmental impact, and management issues after the outbreak of COVID-19: A case study of the Hubei Province in China
Source: PLoS One. 2022 Jan 24;17(1):e0259207. doi: 10.1371/journal.pone.0259207 (PMC8786120; doi:10.1371/journal.pone.0259207)
Supplement: S1 File — All publicly available data are in tabular form in this document. (DOCX) [file pone.0259207.s001.docx]

**Supplementary Information for:**

**Assessment of medical waste generation, associated environmental impact, and management issues after the outbreak of COVID-19: a case study of the Hubei Province in China**
Short title: Medical waste management after COVID-19 in Hubei Province, China

Yifan Song^1^^†^, Jinquan Ye^2†^, Yurong Liu^3^, Yun Zhong^1^*

*^1^Ji luan Academy, Nanchang University, Nanchang 330031, PR China*

*^2^School of Management, Nanchang University, Nanchang 330031, PR China*

*^3^School of Economics and Management, Nanchang University, Nanchang 330031, PR China*

*Corresponding author: *Expertyun@gmail.com*

† *Yifan Song and Jinquan Ye contributed equally to this w**ork*

#### Seasonal-ARIMA model

The figure below shows the seasonal variation of medical waste, as seen in the rose diagram: medical waste tends to be lower in volume in February and higher in volume in March or July. The movement of the edge line of the rose diagram also reflects the elevation of the quantity of medical waste generated over time.


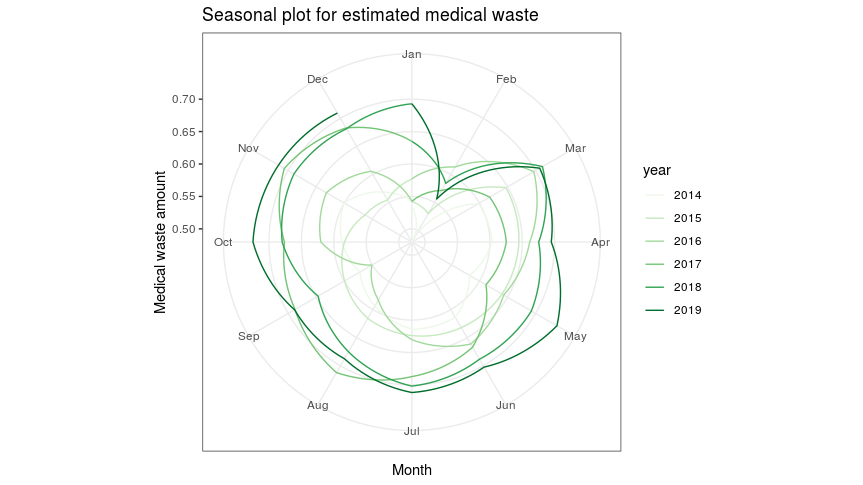


Fig a Monthly Medical Waste Rose Chart

According to its periodic fluctuations, a SARIMA model is adopted for fitting, using 84% of the data as the training set and the remaining 16% as the validation set, and a stepwise regression is used to build the SARIMA$\left( 1,1,1 \right)\left( 1,0,0 \right)$ model.


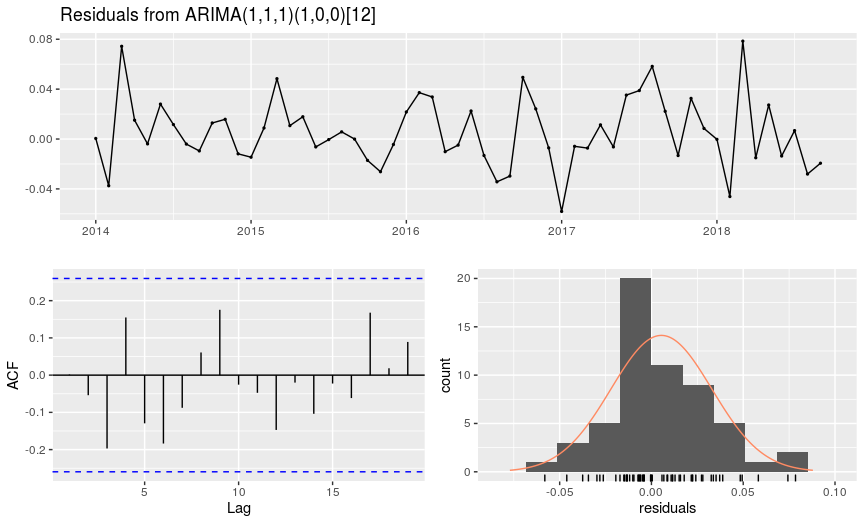
Checking the residual terms of this model, there is no obvious autocorrelation, which meets the white noise requirement, and the fit of this model is acceptable.

Fig b Fitting test

It is made to predict the validation set data and obtain the predicted values with the prediction confidence intervals, and the results are shown in the following figure.


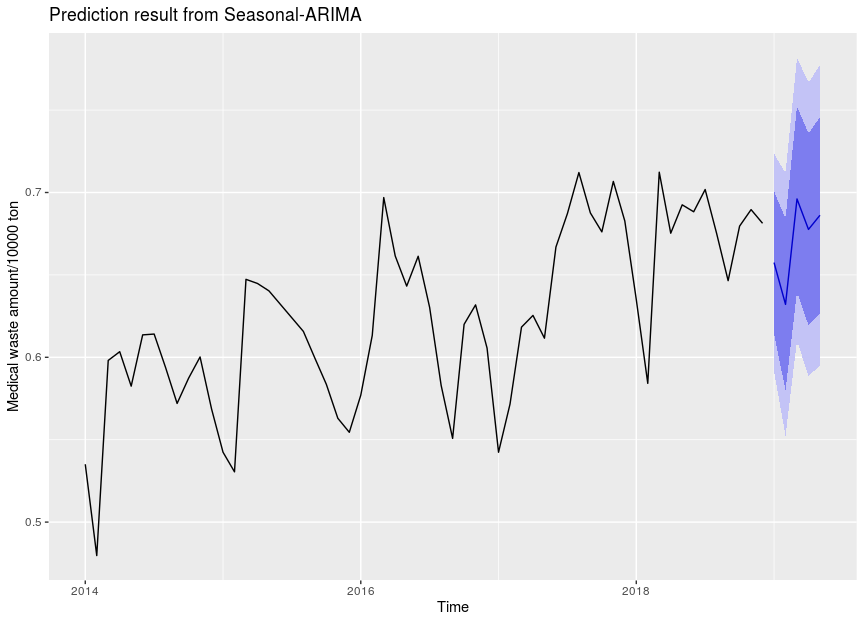


Fig c ARIMA model test set predictions

#### LSTM model


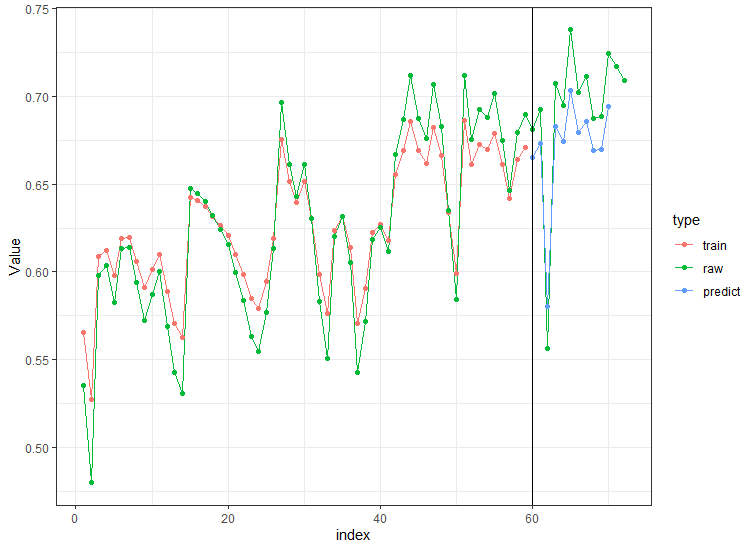
The LSTM model was then used to predict the same data set and to compare the difference in performance between the two. The number of hidden layer neurons was set to 40, the number of hidden layers was set to 1, the number of recursive steps folded in one time step was set to 1, and the number of training rounds was 2000. the AdaGrad algorithm (adaptive gradient algorithm) was used for learning, and the learning rate was 0.7. the fitting results were as follows.

Fig d LSTM fitting effect

The green curve represents the actual curve, the red curve is the fitted training curve, and the blue curve is the prediction of the results for the validation interval, with the predicted values on the vertical axis, which shows that the LSTM model achieved a better fitting effect.

#### Prophet Model

Since Figure 1demonstrates seasonal variation with non-fixed cycles, the Prophet model is also used for prediction in this paper, using the same training and test sets as in the two examples above and comparing them with the two models mentioned above.


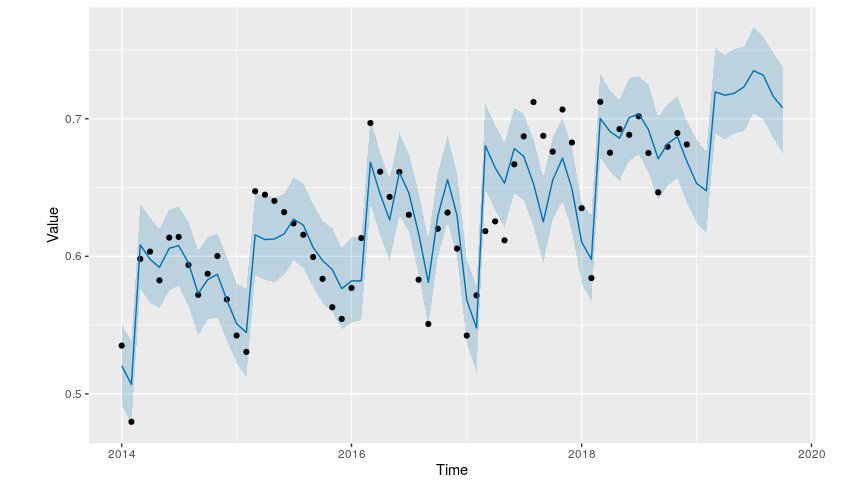
The results are shown in Fig e, where the black color indicates the original time series discrete points, the dark blue line indicates the values obtained by fitting with the time series, and the light blue area indicates the confidence interval of the time series. At the end of the curve, the original time discretization points disappear, leaving only ten predicted values and their upper and lower bounds.

Fig e Annual trends and cycle changes in medical waste production in Hubei Province

Fig f Prophet model predictions


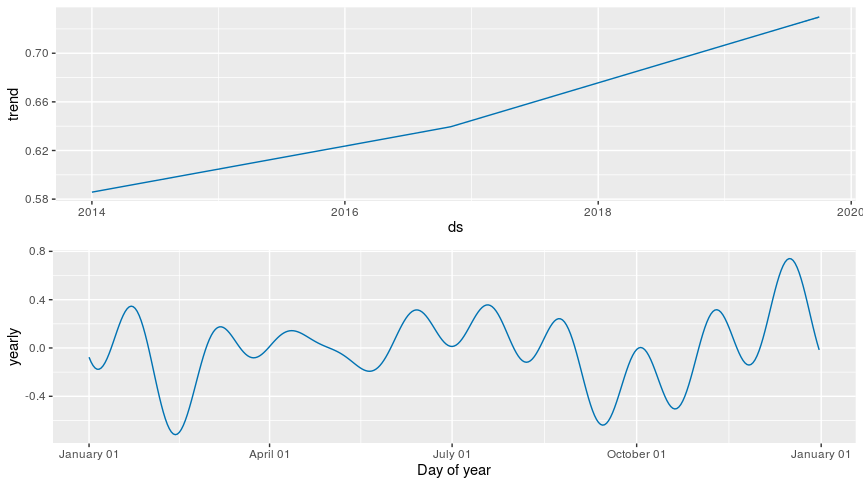


The above figure reflects the decomposed trend term and cycle term, where the upper side of the picture is the trend line, which shows the rising trend of medical waste over time, and the lower side is the annual cycle term, which can be seen in the monthly and daily changes with some regularity.

#### Model Comparison

In this paper, the above models are evaluated using the following five metrics: ^R2^ (Squared Correlation), MAE (Mean Absolute Error), MSE (Mean Square Error), RMSE (Root Mean Square Error) and MAPE ( Average absolute percentage error). These metrics are currently the classical metrics for regression prediction in machine learning and are calculated as follows.

$$\begin{aligned} MAE (y,\hat{y})=\frac{1}{n}\sum_{i=1}^{n} \left| y_{i}-\hat{y}_{i} \right|\#(8) \end{aligned}$$

$$\begin{aligned} MSE (y,\hat{y})=\frac{1}{n}\sum_{i=1}^{n} \left( y_{i}-\hat{y}_{i} \right)^{2}\#(9) \end{aligned}$$

$$\begin{aligned} RMSE (y,\hat{y})=\sqrt{\frac{1}{n}\sum_{i=1}^{n} \left( y_{i}-\hat{y}_{i} \right)^{2}}\#(10) \end{aligned}$$

$$\begin{aligned} R^{2}(y,\hat{y})=1-\frac{\sum_{i=0}^{n} \left( y_{i}-\hat{y}_{i} \right)^{2}}{\sum_{i=0}^{n} \left( y_{i}-\bar{y} \right)^{2}}\#(11) \end{aligned}$$

Using the above metrics, the dispersion of the sample error can be seen in relation to the explanatory strength of the model, and we expect the model to be as low as possible in the MAE, MSE, and RMSE terms, while the$R^{2}$ terms as high as possible. The above model is evaluated, where the goodness of the prediction result indicator is indicated by the color of the table, and the darker the color means the better the effect of that indicator, as shown in the following table.

Table A Model comparison

| Evaluation indicators* | LSTM | SARIMA | Prophet |
| --- | --- | --- | --- |
| MAE | -0.01914899 | -0.01591894 | 0.01659451 |
| MSE | 0.0005968977 | 0.001363096 | 0.001493831 |
| RMSE | 0.02443149 | 0.03692014 | 0.0386501 |
| ^R2^ | 0.9999902 | 0.727125 | 0.4578706 |

It can be seen that the LSTM model performs well on most of the indicators, while the Prophet model is less adaptive and the Seasonal-ARIMA model performs generally, according to which, this paper will use the LSTM for counterfactual forecasting of medical waste in 2020.

By bringing in the LSTM model, the counterfactual prediction for 2020 when no new crown epidemic occurs is obtained: the medical waste generation rate at 0.5 kg/bed/day case, the medical waste generation from January to April 2020 is 0.6765, 0.5838, 0.6864 and 0.6777 tons, respectively. Meanwhile, for the completeness of the results, this paper also predicts the medical waste generation rate at 0.4 kg/bed/day vs. 0.6kg/bed/day in the following figure.


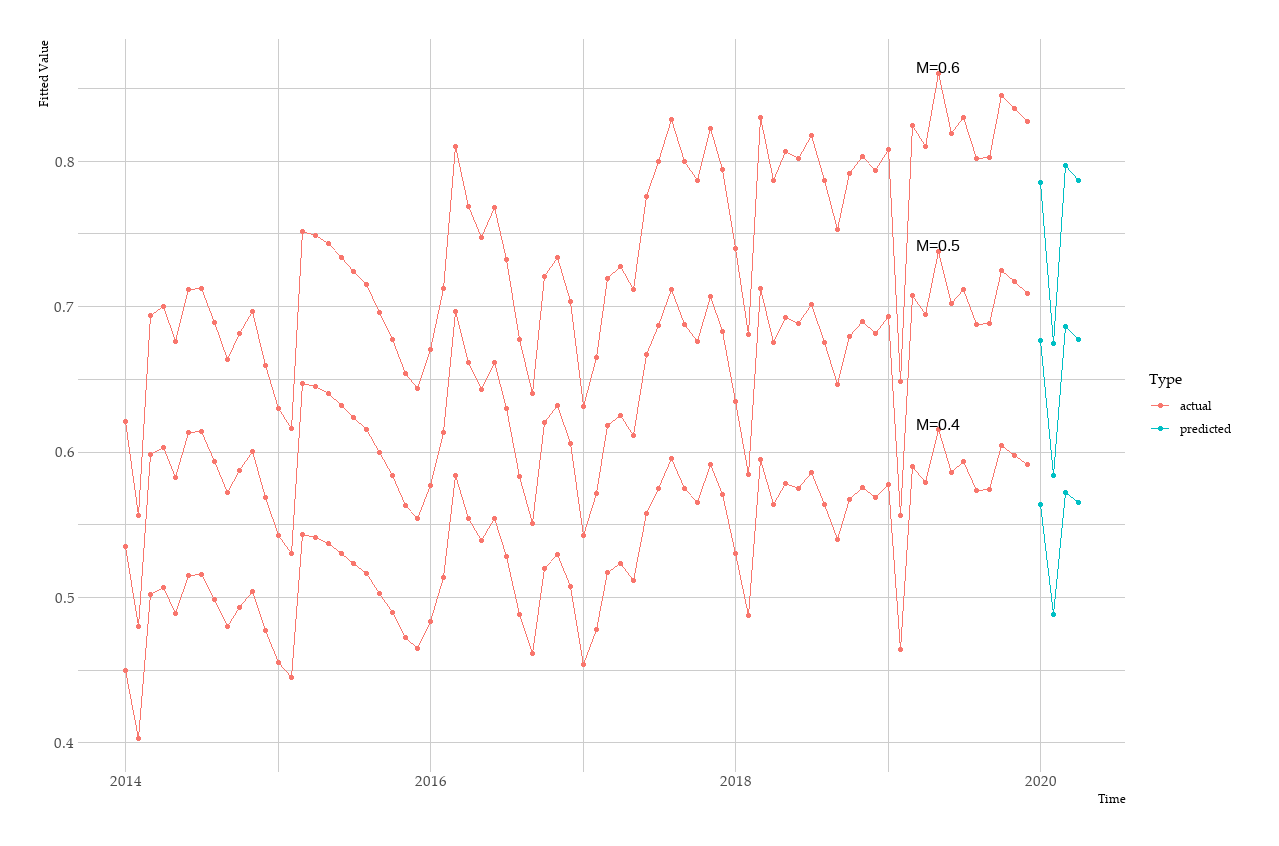


Fig g LSTM prediction results

where Index indicates the temporal order of the series, from 0 to 72 corresponding to a total of 72 observations from January 2014 to December 2019. The blue curve (raw) represents the actual curve and the red curve (predict) is the predicted curve, in which the LSTM predicts the values of the sequence for the next four months.

#### Environmental Impact Assessment Data (BAU)

Table B Pollutant emission values under BAU conditions

|  | Substance | Discharge amount(M=0.4) | Discharge amount(M=0.5) | Discharge amount(M=0.6) |
| --- | --- | --- | --- | --- |
| Wastewater | | 20434.26$m^{3}$ | 41524.04$m^{3}$ | 115.25$m^{3}$ |
| Direct air emissions | Sulfur dioxide | 1213.08$kg$ | 3.37$kg$ | 596.97$kg$ |
|  | Nitrogen oxides | 1161.77$kg$ | 3.22$kg$ | 571.71$kg$ |
|  | Carbon monoxide | 2170.28$g$ | 6.02$g$ | 1068.01$g$ |
|  | Hydrogen fluorid | 8222.02$g$ | 22.82$g$ | 4046.11$g$ |
|  | Hydrogen chloride | 10630.40$kg$ | 29.50$kg$ | 5231.29$kg$ |
|  | Copper | 3881.46$g$ | 10.77$g$ | 1910.09$g$ |
|  | Lead | 125.21$mg$ | 0.35$mg$ | 61.62$mg$ |
|  | Nickel | 25041.68$g$ | 69.50$g$ | 12323.18$g$ |
|  | Cadmium | 2128.54$mg$ | 5.91$mg$ | 1047.47$mg$ |
|  | Chromium | 15609.32$g$ | 43.32$g$ | 7681.45$g$ |
|  | Manganese | 3881.46$g$ | 10.77$g$ | 1910.09$g$ |
|  | Tin | 834.72$mg$ | 2.32$mg$ | 410.77$mg$ |
|  | Mercury | 15451.95$mg$ | 42.89$mg$ | 7604.01$mg$ |
|  | Antimony | 20617.65$mg$ | 57.22$mg$ | 10146.09$mg$ |
|  | Dioxins (TEQ) | 1043.40$\mu g$ | 2.90$\mu g$ | 513.47$\mu g$ |
|  | Particulates | 15775.58$g$ | 43.79$g$ | 7763.27$g$ |
|  | Carbon dioxide | 525.46$t$ | 1.46$t$ | 258.58$t$ |
|  | Ammonia | 747.15$g$ | 2.07$g$ | 367.68$g$ |
| Direct soil emissions | Ammonia | 373.57$g$ | 1.04$g$ | 183.84$g$ |
|  | Mercury | 26.68$mg$ | 0.07$mg$ | 13.13$mg$ |
|  | Phenol | 54941.86$mg$ | 152.49$mg$ | 27037.27$mg$ |
|  | Chromium | 5790.38$mg$ | 16.07$mg$ | 2849.48$mg$ |
|  | Chloride | 20893.38$g$ | 57.99$g$ | 10281.78$g$ |

Table C Life cycle inventory of each scenario. Values are presented per functional unit

| Substance | | Unit | Amount | | |
| --- | --- | --- | --- | --- | --- |
|  |  |  | Pyrolysis | Chemical disinfection | Steam sterilization |
| Raw materials | Fresh water | t | 2.9 | 0.48 | 2.14 |
|  | Land occupation | m2 | 0.09 | 0.1 | 0.08 |
|  | Lime | kg | 34 | 0.07 | - |
|  | Organic chemicals | g | - | 3.75 | - |
|  | Sodium hydroxide | t | 0.17 | - | - |
|  | Sodium hypochlorite | kg | 2.78 | - | - |
|  | Chlorine dioxide | kg | - | 3.76 | - |
|  | Chlorine | kg | - | - | 0.37 |
|  | Activated carbon | kg | 13 | 2.42 | 0.26 |
|  | Diesel | kg | 8.8 | - | 48.09 |
|  | Electricity | kWh | 238.89 | 420.61 | 774.97 |
| Solid Waste | Sanitary landfill | t | 0.06 | 1.08 | 1.02 |
|  | Hazardous waste incineration | kg | - | 4.68 | 0.26 |
| Wastewater |  | m3 | 5.64 | 1.31 | 6.74 |
| Direct air emissions | Sulfur dioxide | kg | 0.15 | - | 0.22 |
|  | Nitrogen oxides | kg | 0.24 | - | 0.06 |
|  | Carbon monoxide | g | 0.52 | - | - |
|  | Hydrogen fluoride | g | 1.97 | - | - |
|  | Hydrogen chloride | kg | 0.45 | - | 3.28 |
|  | Copper | g | 0.93 | - | - |
|  | Lead | mg | 0.03 | - | - |
|  | Nickel | g | 6 | - | - |
|  | Cadmium | mg | 0.51 | - | - |
|  | Chromium | g | 3.74 | - | - |
|  | Manganese | g | 0.93 | - | - |
|  | Tin | mg | 0.2 | - | - |
|  | Mercury | mg | 3.6 | - | 0.16 |
|  | Antimony | mg | 4.94 | - | - |
|  | Dioxins (TEQ) | mg | 0.25 | - | - |
|  | Particulates | g | 0.11 | 23.33 | 5.74 |
|  | Carbon dioxide | t | 0.03 | - | 0.15 |
|  | Ammonia | g | - | 0.43 | 0.28 |
|  | Volatile organic compounds | g | - | 5.57 | - |
| Direct soil emissions | Ammonia | g | - | 1.78 | 0.14 |
|  | Petroleum oil | mg | - | - | 43.48 |
|  | Mercury | mg | - | - | 0.01 |
|  | Biogenic oil | g | - | - | 1.67 |
|  | Phenol | mg | - | - | 20.59 |
|  | Chromium | mg | - | - | 2.17 |
|  | Chloride | g | - | 0.1 | 7.83 |
|  | Phosphorus | mg | - | 12.79 | - |

#### Related Toxicology List Information

Table D Toxicological information and regulatory provisions of relevant toxicants^^[[1]](#footnote-1)^^[[2]](#footnote-2)^^

| Number | Name | Analysis methods | Standard number | Emission limit values  (Class Ⅱ areas) | Source | Hazards |
| --- | --- | --- | --- | --- | --- | --- |
| 1 | Carbon  Monoxide | Air quality Determination of carbon monoxide Non-dispersive infrared method | GB 9801 | 4 mg/$\text{m}^{3}$  (24 hour average) | Heating equipment mainly for wards and operating rooms | Causes symptoms such as impaired mental state and difficulty in breathing |
| 2 | Nitrogen  oxides | Determination of nitrogen oxides (nitrogen monoxide and nitrogen dioxide)in ambient air Saltzman method | HJ 479 | 0.01mg/$\text{m}^{3}$  (24 hour average) | Mainly for power generation and heating equipment in hospitals | Increased coughing and wheezing, which can easily lead to airway inflammation |
| 3 | Fluoride | Determination of mass concentration of fluoride in ambient air Filter membrane - fluoride ion selective electrode method | GB/T 15434-1995 | 0.5mg/$\text{m}^{3}$  (24 hour average) | Mainly from antirust agents and pharmaceutical reagents in hospitals | Irritates the body's eyes and throat and other organs and may cause respiratory bleeding |
| 4 | Sulphur  dioxide | Determination of Sulfur Dioxide in Ambient Air Formaldehyde Absorption-Para Rose Aniline Spectrophotometry | HJ/482 | 0.015mg/$\text{m}^{3}$  (24 hour average) | Mainly from the hospital's power generation, heat generation equipment and metal processing equipment | Causes respiratory symptoms and reduces lung function |
| 5 | Mercury | Cold Atomic Absorption Method of Analysis for Air and Exhaust Gas Monitoring National Environmental Protection Agency (1990) | / | 0.05mg/$\text{m}^{3}$  (annual average) | Mainly from thermometers, blood pressure cuffs, thermostats, fluorescent lamps and other products used in hospitals | Mercury poisoning tends to cause symptoms such as weakened memory, attention span, weakened vision, and muscle weakness |
| 6 | Dioxins | Water quality Dioxin determination Isotope dilution High-resolution gas chromatography-high-resolution mass spectrometry | HJ77.1-2008 | 0.5 ng TEQ/N$\text{m}^{3}$ (annual average) | Almost all incineration of medical waste produces large amounts of dioxins | Causes skin damage and many types of cancer in humans |
| 7 | Acetaldehyde | Determination of acetaldehyde in exhaust from stationary sources Gas chromatography method | HJ/T35-1999 | 4× 10-2 mg/$\text{m}^{3}$  (annual average) | Mainly from building materials and disinfectants in hospitals, electrocautery fumes in operating theatres etc. | Prone to cardiovascular disease, cancer, stroke, coronary heart disease and other diseases |
| 8 | Formaldehyde | Air quality Determination of formaldehyde Acetylacetone spectrophotometric method | GB/T15516-1995 | 0.5 mg/$\text{m}^{3}$  (annual average) | Mainly from building materials and disinfectants in hospitals, electrocautery fumes in operating theatres etc. | Prone to cardiovascular disease, cancer, stroke, coronary heart disease and other diseases |
| 9 | Aniline | Air quality Determination of aniline Naphthylenediamine hydrochloride spectrophotometric method | GB/T 15502-1995 | 0.5~600 mg/$\text{m}^{3}$  (annual average) | Mainly from antimicrobial and chemotherapeutic agents in hospitals | It can cause allergic reactions in contact with human skin and can cause poisoning and carcinogenesis when it enters the body |
| 10 | Organic  sulphides | Gas Chromatography Method of Analysis for Air and Exhaust Gas Monitoring" National Environmental Protection Agency (1990) | / | 2 mg/$\text{m}^{3}$  (annual average) | Mainly from the process of decomposition of large amounts of organic material in the hospital | May irritate the mucous membranes of the eyes, causing headaches, nausea and memory loss |
| 11 | O3  (Ozone) | Determination of ozone in ambient air Sodium indigo diflavinate spectrophotometric method | HJ 504 | 0.2 mg/$\text{m}^{3}$  (annual average) | Produced by the photochemical reaction of nitrogen oxides and volatile organic compounds (VOCs) | Ozone can strongly irritate the respiratory tract, causing sore throats, bronchitis and emphysema, and can even cause neurotoxicity |

Notes:

1. Classification of ambient air functional areas.

Ambient air functional areas are divided into two categories: Class I areas are nature reserves, scenic spots and other areas requiring special protection; Class II areas are residential areas, mixed commercial and traffic residential areas, cultural areas, industrial areas and rural areas. Here, because of the study of medical waste, it is designated as a Class II area.

2. normative citation documents.

This standard cites the following documents or articles therein. Where the cited documents are not dated, their latest versions are applicable to this standard.

GB 8971 Determination of benzo[a]pyrene in air quality drifting dust ﹑ acetylated filter paper chromatography fluorescence spectrophotometric method.

GB 9801 air quality carbon monoxide determination of non-dispersive infrared method.

GB/T 15264 ambient air lead determination of flame atomic absorption spectrophotometry GB/T 15432 ambient air total suspended particulate matter determination of weight method.

GB/T 15439 ambient air benzo[a]pyrene determination of the high performance liquid chromatography method.

HJ 479 ambient air nitrogen oxides (nitric oxide and nitrogen dioxide) determination, naphthalene diamine hydrochloride spectrophotometric method HJ 482 ambient air sulfur dioxide determination of formaldehyde absorption-para rose aniline spectrophotometric method.

HJ 483 ambient air sulfur dioxide determination of the four chlorine mercury salt absorption - vice rose aniline spectrophotometric method HJ 504 ambient air ozone determination of sodium indigo bisulfate spectrophotometric method.

HJ539 ambient air to know the determination of lead to know graphite furnace atomic absorption spectrophotometry (provisional) HJ590 ambient air ozone to know the determination of ultraviolet photometric method.

HJ618 ambient air PMo and PM.s determination of the weight method HJ 630 environmental monitoring quality management technical guidelines.

HJ/T 193 ambient air quality automatic monitoring technical specification HJ/T 194 ambient air quality manual monitoring technical specification.

Ambient Air Quality Monitoring Specifications (Trial)" (State Environmental Protection Administration Announcement No. 4 of 2007).

Guiding Opinions on Promoting Joint Prevention and Control of Air Pollution to Improve Regional Air Quality (Guo Ban Fa [2010] No. 33).

3. fluoride.

Refers to inorganic fluoride in gaseous and particulate form.

24-hour average.

Refers to the arithmetic average of the 24-hour average concentration of a natural day, also known as the daily average.

Annual average.

The arithmetic mean of the average daily concentrations in a calendar year.

1. Emission limit values and basis method reference website. http://english.mee.gov.cn/Resources/standards/Air_Environment/quality_standard1/201605/t20160511_337502.shtml [↑](#footnote-ref-1)
2. Source and Hazard Reference.https://www.epa.gov/haps/health-effects-notebook-hazardous-air-pollutants [↑](#footnote-ref-2)
