# Supplementary material for: Assessment of medical waste generation, associated environmental impact, and management issues after the outbreak of COVID-19: A case study of the Hubei Province in China
Source: PLoS One. 2022 Jan 24;17(1):e0259207. doi: 10.1371/journal.pone.0259207 (PMC8786120; doi:10.1371/journal.pone.0259207)
Supplement: S2 File — (PDF) [file pone.0259207.s002.pdf]

Confirmation of reordering authorship

The authors agreed on the change of author order: Jinquan Ye changed to the first author of this paper.

Written confirmation:

Jinquan Ye

Yun Zhong

Yifan Song

Yurong Lou
